# Confocal Laser Endomicroscopy in Neurosurgery: A New Technique with Much Potential

**DOI:** 10.1155/2013/851819

**Published:** 2013-07-28

**Authors:** David Breuskin, Jana DiVincenzo, Yoo-Jin Kim, Steffi Urbschat, Joachim Oertel

**Affiliations:** ^1^Department of Neurosurgery, Saarland University, 66421 Homburg, Germany; ^2^Department of Pathology, Saarland University, 66421 Homburg, Germany

## Abstract

Technical innovations in brain tumour diagnostic and therapy have led to significant improvements of patient outcome and recurrence free interval. The use of technical devices such as surgical microscopes as well as neuronavigational systems have helped localising tumours as much as fluorescent agents, such as 5-aminolaevulinic acid, have helped visualizing pathologically altered tissue. Nonetheless, intraoperative instantaneous frozen sections and histological diagnosis remain the only method of gaining certainty of the nature of the resected tissue. This technique is time consuming and does not provide close-to-real-time information. In gastroenterology, confocal endoscopy closed the gap between tissue resection and histological examination, providing an almost real-time histological diagnosis. The potential of this technique using a confocal laser endoscope EndoMAG1 by Karl Storz Company was evaluated by our group on pig brains, tumour tissue cell cultures, and fresh human tumour specimen. Here, the authors report for the first time on the results of applying this new technique and provide first confocal endoscopic images of various brain and tumour structures. In all, the technique harbours a very promising potential to provide almost real-time intraoperative diagnosis, but further studies are needed to provide evidence for the technique's potential.

## 1. Introduction

Neurooncological diagnosis and treatment constitute a major part of neurosurgery. Obtaining histological diagnosis is frequently challenging. The resulting therapeutic options vary depending on the histological grade and the tumour type. The incidence of gliomas is expected to be around 5-6/100000 per year, with their survival rate depending heavily on their WHO grade. Even with today's high medical standards consisting of surgical removal and postoperative combined radiochemotherapy, median survival shows 18–21 months at its best for glioblastomas [1]. While it is frequently noted that malignant gliomas cannot be cured by surgical resection, recent studies show an improved life expectancy associated with a more extended tumour resection [1–3]. Thus, currently, research is focussing on increasing the extent of resection through various additional techniques such as neuronavigation [4] or 5-aminolaevulinic acid (5-ALA) fluorescent marking of tumour cells. While neuronavigation suggests precise imaging of the tumour, this can be misleading due to brain shift occurring during surgery and therefore tumour borders are not depicted according to reality [5]. For 5-ALA, randomized clinical trials showed a significant reduction of second resection in patients treated with 5-ALA compared to those who had surgery being performed solely under white light [6]. However, not all tumour cells show fluorescent activity; thus, neither the introduction of neuronavigation nor 5-ALA tumour imaging solved the problem of intraoperative precise separation of tumour tissue from adjacent intact brain parenchyma.

A new way of optical imaging is confocal laser endomicroscopy (CLE) which has recently been applied to other medical fields such as gastroenterology and pulmonology. As a patent dating back to 1957, confocal microscopy manages to reduce emitted light by molecules that are not in the desired focus plane. Opposed to conventional fluorescence microscopes where the tissue is widely lit upon, confocal laser microscopy only emits a punctual light beam from a laser source reducing the amount of scattered light that is then emitted by the sample. Because of an interposed pinhole blocking all remaining scattered light, only light emitted by the desired point is detected. The confocal light generates clear focused images without any out of focus signals. This technique has allowed visualizing the underlaying tissue on a microscopic scale with its features notably depending on the device in use. Through this method, however, it has been possible to achieve real-time imaging on a scale that has previously only been possible on histologic slices, making it a powerful diagnostic tool for tissue alterations. In gastroenterology as well as in pulmonology, the technique has been used in a combined method with standard endoscopy, giving the possibility of microscopic evaluation combined with targeted biopsies of altered tissue [7–9]. 

With these promising results, CLE was introduced to neurosurgery and is currently being evaluated in different settings. It is a common goal that this technique can be used in an easy intraoperative setting, allowing neurosurgeons to scan tumour borders, allowing for more precise resections to be made and improving the outcome of patients with brain tumours.

## 2. Materials and Methods

### 2.1. General Study Design

The application of a confocal endomicroscope (EndoMAG1) manufactured by KARL Storz company, Tuttlingen, Germany, on human tumour specimen and human tumour cell cultures in order to analyse the value of this device in neurooncology was investigated.

### 2.2. Confocal Endoscope

**Figure 1:**
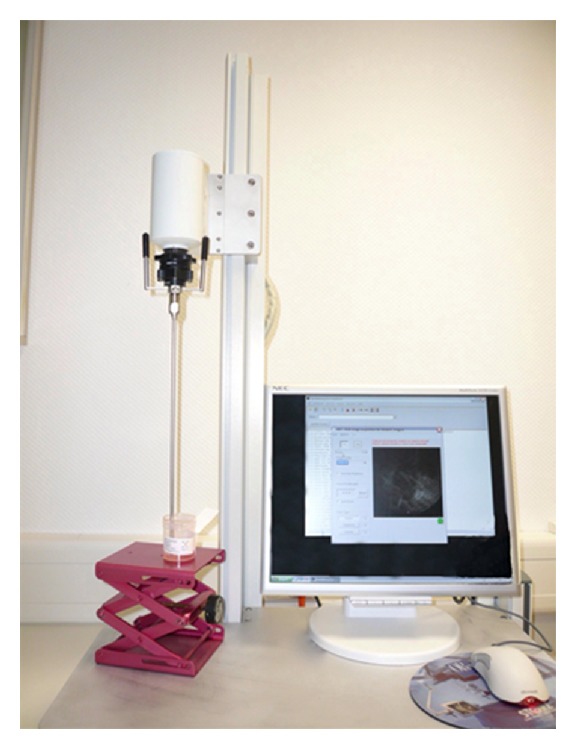
Confocal endomicroscope EndoMAG1.

The imaging device comprises of a rigid endoscope with Hopkins-rod lenses mounted on a fixed frame connected to the imaging device and computer. The outer diameter is 5 mm, and the length amounts to 323 mm. The size of the circular scanning field covers 300 *μ*m × 300 *μ*m, and the highest achievable resolution is 2 *μ*m. The wavelength of the laser signal is red, and scanning depth in 3D mode is approximately 80 *μ*m. The detected signal consists of reflection and scattering. The frame rate (2D) is almost 40 frames per second allowing true real-time images to be evaluated. The setting of the CLE does not yet allow the investigation at location during surgery. Tissue samples had to be removed first and taken to the work station depicted in Figure 1 in order to be examined.

### 2.3. Tissue Investigation and Data Evaluation

In the first step, pig brain tissue was used to evaluate general handling aspects and to develop an algorithm to proceed with the tissue samples for optimal CLE results.

In the second step, samples of resected tumour tissue or primary cell cultures were covered in isotonic saline solution as a thin fluid layer improved image quality. The rigid endoscope was then placed on top of the sample, while a slight pressure to the tissue needed to be applied to ensure contact. All tissue samples were then investigated a second time after staining with methylene blue after incubation time of 20 minutes. Methylene blue is an in vivo as well as in vitro staining agent that is safe to use and of no toxic nature to the patient. In histology, it stains nuclei, making their examination favourable. Other than this histological use, MB serves as a spray dye in gastroenterologic endoscopic procedures in order to visualise altered tissue. After starting the software, images of the samples were viewed in real time. Samples were brought in focus by changing the height of the platform. When a clear image was achieved, the tissue was scanned by using the focus on the endoscope. These images were digitally saved and compared to their respective histological slices made by the neuropathologist. All three groups of images, tumour tissue samples, cell cultures, and histological slices were used to define similarities in respect to their original tumour entity, which focused mainly on cell shape and density, shape of the nuclei, and interstitial structures.

## 3. Results and Discussion

**Figure 2:**
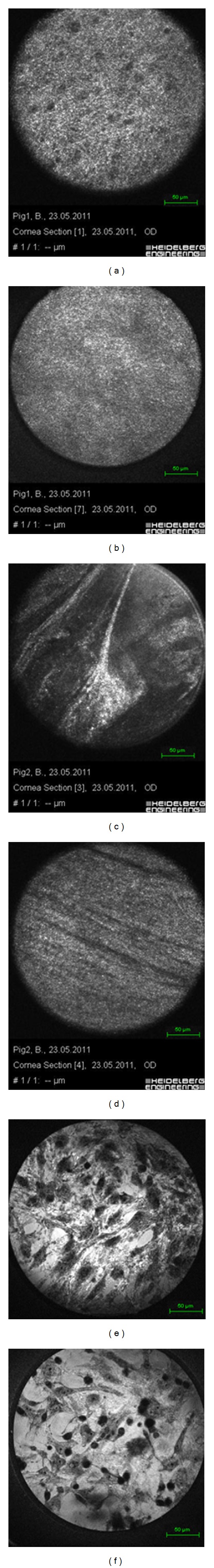
(a) Grey matter of the pig. (b) White matter of the pig. (c) Arachnoid membrane of the pig. (d) Ventricular wall of the pig. (e) Primary meningioma cell culture. (f) Primary glioblastoma cell culture.

In this first trial using this specific device, images of unaltered pig brain tissue and of primary cell cultures were evaluated. It was possible to see structures on an endomicroscopic level detecting different cell structures on a highly focused plane. Additionally, different tissue structures, differences between grey and white substances, and arachnoid membranes could be visualized although the scanning field consisted only of 300 *μ*m × 300 *μ*m (Figures 2(a)–2(f)).

The preparation of the tissue before examination proved to be without difficulties. The samples needed no more than a small layer of liquid—in this series isotonic sodium chloride solution—to improve image quality. Compared to frozen sections done by the neuropathologist, this technique offers a quicker preparation and faster visualisation since staining does not necessarily need to be done. 

Images of the grey substance, for example, showed a higher density in nuclei compared to white substance, giving impressions of the different structures and scale. The tissue structure was much denser compared to arachnoid mater with a more fibrous pattern including elongated cell bodies and fibrillar cytoplasm.

After this first examination, samples were partly stained with methylene blue (MB). For this, the tissue was simply put into MB solution. Depending on the size of the sample, it was best to divide the tissue in small pieces to create a larger contact surface for staining. After 20 minutes of incubation, analysis was performed likewise to the native examination. Results were mainly not very different from native samples. However, the application of MB helped in the evaluation of the nuclei in selected cases since the nuclei presented themselves darker with a more pronounced contrast depending on how well MB had been absorbed. For further investigation as well as intraoperative use, however, the need of MB staining seems to be questionable, as no significant benefit could be observed.

In the second step of analysis, more than 50 tumour specimens were evaluated. It was found that common histological paradigms could not entirely be applied for tumour tissue evaluation. Different endomicroscopic histological criteria were established which were found to be reoccurring amongst different tumour entities. These criteria mainly included the morphology of the nucleus and its location within the cell, the existence and the shape of the cytoplasm, the presence of psammoma bodies, the cell-to-cell contact, the cellular density within the specimen, the growth pattern (i.e., diffuse, well sorted), and the presence of blood vessels. 

**Figure 3:**
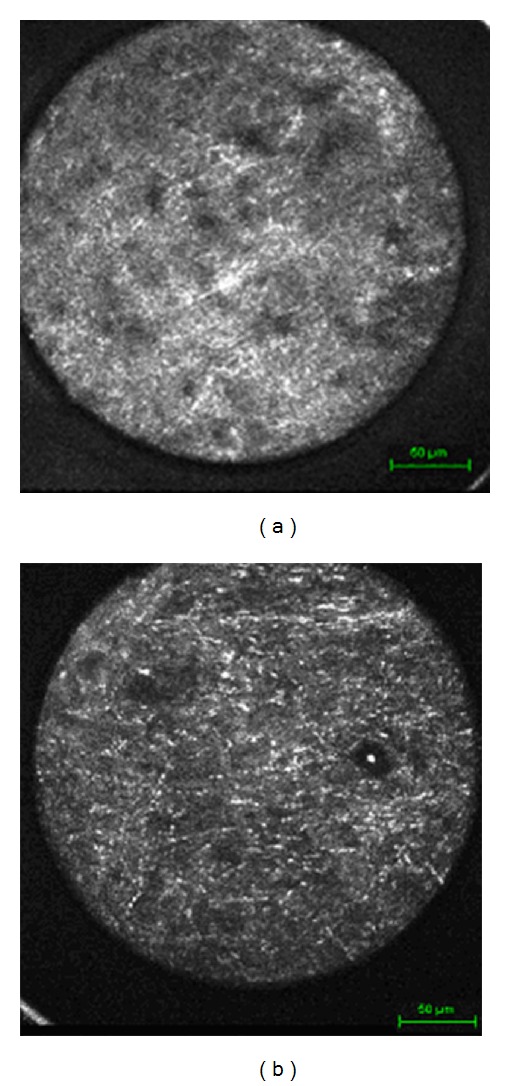
(a) Glioblastoma. (b) Astrocytoma.

Confocal endomicroscopically, glioblastomas showed similarities to normal brain tissue although presenting a higher cellularity. Nuclei were mostly polymorphous and variable in shape, much of how they present themselves in histological findings. However, the most striking confocal diagnostic criterion was a very diffuse growth pattern with cell borders hardly being visible and fibrillar cytoplasm being less remarkable as in low grade gliomas or in normal tissue (Figure 3(a)). In low grade gliomas, cell borders showed a much sharper contrast and more definite glia-like structure (Figure 3(b)).

**Figure 4:**
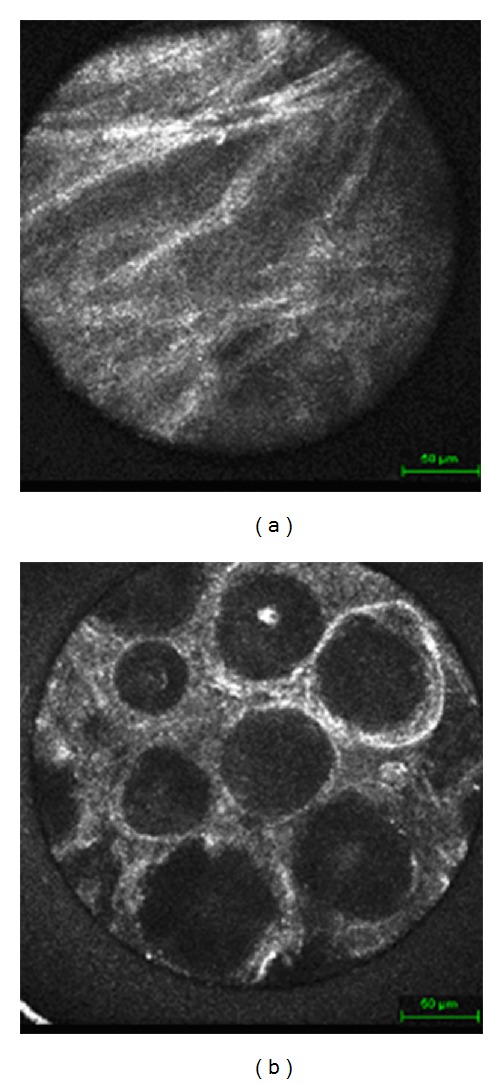
(a) Meningioma. (b) Psammomatous meningioma.

**Figure 5:**
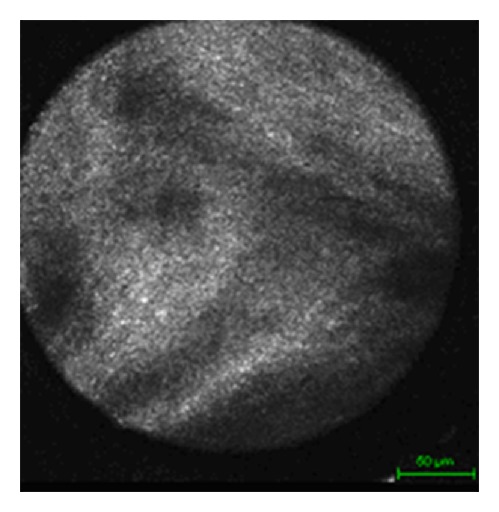
Schwannoma.

Meningiomas showed a very distinct image. Their origin being arachnoid cells, a very well distinguishable fibrous network with oval shaped nuclei and elongated spindle-like cytoplasm, was found (Figure 4(a)). This structure became even more apparent when scanning through the tissue using the focus. An even more precise diagnosis could be made in cases of psammomatous meningiomas when characteristic psammoma bodies were present and scattered throughout the samples (Figure 4(b)). Schwannomas resembled meningiomas in many ways but showed larger fibrous streaks (Figure 5).

As quintessence of this first evaluation of a new confocal laser endoscope, some peculiar aspects can be already summarised and have to be discussed. 

Based on the results in the pig brain and on human tumour cell culture as well as based on the results of fresh human tumour specimen, brain cell and tissue as well as tumour specimen show a very characteristic appearance in confocal endoscopic imaging. Thus, at first sight, confocal endoscopy could provide almost real-time diagnosis of human brain tumours. But further studies are needed before any conclusions can be made.

These results reflect some of the aspects mentioned by other groups using confocal endomicroscopic techniques [10–12]. While the devices in use differ, examination of tumorous tissue provides images that allow a histological differentiation from healthy brain tissues. With the EndoMAG1, however, no fluorescent agents were needed in order to investigate the probes, which ultimately makes intraoperative use easier and, in cases of toxic agents, safer for patients.

Intraoperative detection of tumour margins as well as identification of altered cerebral tissue is one of the most demanding aspects of brain tumour surgery. Improving the quality of the surgical procedure through much technical advancement throughout the past recent years, operative visualisation still has many downsides. High grade gliomas infiltrate the tissue that seems unaltered under the surgical microscope, which is why many tumours cannot be radically removed yet. Confocal laser endomicroscopy is aiming to close this gap between molecular imaging and surgical microscopic imaging. Introduced and well established, the technique might very well have the potential to change the surgical strategy by its intraoperative application. The potential of gathering real-time histopathology will eventually help neurosurgeons to thoroughly scan borders of the resection area determining whether an extension of resection is needed. Surgical procedures could then be kept as minimally invasive as possible while removing as much as possible without causing neurologic damage because of excessive resection.

CLE is yet to be properly investigated in order to be fully integrated into standard neurosurgical procedures, but few groups are currently evaluating different devices as well as techniques, all of them benefiting from the knowledge of nonneurosurgical fields of application [10–12]. Further trials will see this device being used on different tumour entities to gather sufficient data for accurate intraoperative histological diagnosis. Whether ordinary histological paradigms are applicable is yet to be examined. It is possible that different criteria need to be found to evaluate the samples as it has been done in gastroenterology [13].

## 4. Conclusion

Confocal laser endoscopy with the EndoMAG1 provides reliable images applied at pig brain, cell tissue cultures, and fresh human brain tumour tissue. All structures seem to harbour a very characteristic endoscopic image. Thus, potentially, this technique could provide a real-time histological diagnosis. But before this even could be discussed, a further development of the endoscope and a detailed analysis of the correlation of confocal endoscopic imaging and histopathological diagnosis have to be done in further studies.
